# HER2 as a target in invasive urothelial carcinoma

**DOI:** 10.1002/cam4.432

**Published:** 2015-02-26

**Authors:** Joaquim Bellmunt, Lillian Werner, Aristotle Bamias, André P Fay, Rachel S Park, Markus Riester, Shamini Selvarajah, Justine A Barletta, David M Berman, Silvia de Muga, Marta Salido, Enrique Gallardo, Federico Rojo, Elizabeth A Guancial, Richard Bambury, Stephanie A Mullane, Toni K Choueiri, Massimo Loda, Edward Stack, Jonathan Rosenberg

**Affiliations:** 1 Bladder Cancer Center, Lank Center for Genitourinary Oncology, Dana-Farber Cancer InstituteBoston, Massachusetts; 2 Department of Medical Oncology, University Hospital de Mar–IMIMBarcelona, Spain; 3 Department of Biostatistics and Computational Biology, Dana-Farber Cancer InstituteBoston, Massachusetts; 4 University of Athens and Hellenic Co-operative Oncology GroupAthens, Greece; 5 Center for Molecular Oncologic Pathology, Department of Pathology, Brigham and Women's HospitalBoston, Massachusetts; 6 Department of Pathology, Brigham and Women's HospitalBoston, Massachusetts; 7 The Johns Hopkins University School of MedicineBaltimore, Maryland; 8 Hospital de Mar Research Institute–IMIMBarcelona, Spain; 9 Hospital Parc TauliSabadell, Spain; 10 IIS–Fundacion Jimenez DiazMadrid, Spain; 11 Memorial Sloan Kettering Cancer CenterNew York City, New York

**Keywords:** ERBB2, genomic alterations, HER2, prognosis, urothelial carcinomas

## Abstract

We evaluated primary tumors from two cohorts, Spain (*N* = 111) and Greece (*N* = 102), for patients who were treated with platinum-based chemotherapy. Patients were tested for HER2 status (IHC score of 3+ or FISH ratio of ≥2.2) by immunohistochemistry (IHC), fluorescence in situ hybridization (FISH), DNA copy number, mRNA expression, and mutation status in patients with metastatic urothelial carcinoma (UC), and its impact on survival. *ERBB2* mutation was determined by hotspot sequencing. mRNA expression was assessed using NanoString counting. Association of overall survival (OS) and HER2 status was assessed by a Cox regression model. NIH-3T3 cells containing HER2 V777L were assessed for growth, invasion, and HER2 kinase activation. In all, 22% of Spanish and 4% of Greek cohorts had 3+ HER2 staining by IHC. FISH amplification was identified in 20% of Spanish and 4% of Greek cohorts. Kappa coefficient between FISH and IHC was 0.47. HER2 status was not associated with OS in univariate (Spanish *P* = 0.34; Greek *P* = 0.11) or multivariate (Spanish *P* = 0.49; Greek *P* = 0.12) analysis. HER2-positive tumors expressed higher levels of HER2 mRNA than HER2-negative tumors (*P *<* *0.001). HER2 mutations (V777L and L755S) were identified in two (2%) patients. In vitro analysis of V777L results in transformation of NIH-3T3 cells, leading to increased growth, invasion on soft agar, and HER2 kinase constitutive activation. In summary, HER2 overexpression or amplification in the primary tumor did not predict OS in patients with metastatic UC. HER2 positivity rates can differ between different populations. Further trials in genomically screened patients are needed to assess HER2-targeted therapies in UC.

## Introduction

Of all patients diagnosed with urothelial carcinoma (UC), roughly 20% will present with metastatic UC, and another 20% will progress to metastatic disease over time, which is nearly uniformly fatal 1. Although untreated UC is frequently chemosensitive, nearly all tumors become resistant to standard platinum-based combination therapies. Unfortunately, the treatment of metastatic UC has not improved significantly in 20 years, in part, due to the lack of validated therapeutic targets beyond cytotoxic agents.

Human epidermal growth factor receptor 2 (HER2) overexpression (encoded by the *ERBB2* gene) has long been a prognostic marker and predictive tool in the treatment of breast cancer, and more recently in esophagogastric cancer 2,3. In breast cancer, overexpression and *ERBB2* DNA amplification are generally closely linked. For specimens with intermediate HER2 protein expression, fluorescence in situ hybridization (FISH) identifies patients which will benefit from HER2-targeted therapies 4.

In addition to amplification and overexpression, mutations in *ERBB2* have been reported in multiple cancer types, including UC, and are oncogenic in vitro 5–8. Recently, mutations in the extracellular domain of *ERBB2* were found to be present in 40% of micropapillary UC 9. Since extracellular domain mutations may confer sensitivity to *ERBB2* kinase inhibitors, these findings may result in new therapeutic opportunities in selected UC patients 7.

Similar to breast cancer, the mechanisms of UC HER2 overexpression include DNA amplification and/or protein overexpression. Reports of HER2 overexpression in UC have demonstrated frequencies of alteration ranging from 6% to 80% 10–18.

Several studies have shown a significantly higher incidence of HER2-positive tumors in advanced disease and metastases, suggesting HER2 may not only be a biomarker for more aggressive disease but also a potential therapeutic target 11,13. However, other studies have found no such association 1,10,11,14–17,19–21. Furthermore, the association between HER2 status and overall survival (OS) in UC remains unclear with published studies providing conflicting results 22–24.

To address these issues, we undertook an analysis of HER2 in bladder cancer in patients who developed metastatic disease, by evaluating immunohistochemical (IHC) staining for HER2, FISH for *ERBB2* on two cohorts of patients, targeted *ERBB2* mutation hotspot sequencing, mRNA expression by NanoString, and *ERBB2* copy number by array-based comparative genomic hybridization (aCHG) in primary tumors from one of these cohorts. For selected hotspot mutations, in vitro evaluation of their oncogenic potential was undertaken in NIH-3T3 cells.

## Methods

### Patients

Patients from two cohorts were used for this analysis. One cohort of 111 patients was obtained from biospecimen banks from three Spanish hospitals (University Hospital del Mar in Barcelona, Hospital Parc Taulí in Sabadell, and Fundación Jimenez Diaz in Madrid). Each patient received platinum-based combination chemotherapy for metastatic disease. The other cohort of 102 consisted of patients treated on a phase III study of dose-dense gemcitabine and cisplatin or dose-dense MVAC (methotrexate/vinblastine/doxorubicin hydrochloride/cisplatin) for metastatic UC, as well as some patients treated with gemcitabine and carboplatin 25. Formalin-fixed paraffin-embedded tissue (FFPE) was collected from prior transurethral resection or cystectomy. All the translational studies were performed using standard protocols in the Cytogenetics Laboratory and the Center for Molecular Oncologic Pathology (CMOP). All cases were collected under Institutional Review Board (IRB)-approved protocols at the different institutions, de-identified and approved for use by the Dana-Farber Cancer Institute IRB.

### Tissue preparation

Slides from FFPE tissue blocks were evaluated by two genitourinary pathologists (D. M. B. and J. A. B.). Tumor-bearing areas were identified, and 0.6-mm cores were taken for tissue microarray (TMA) construction and DNA extraction. Each specimen was represented in triplicate in the TMA.

### Immunohistochemistry

**Figure 1:**
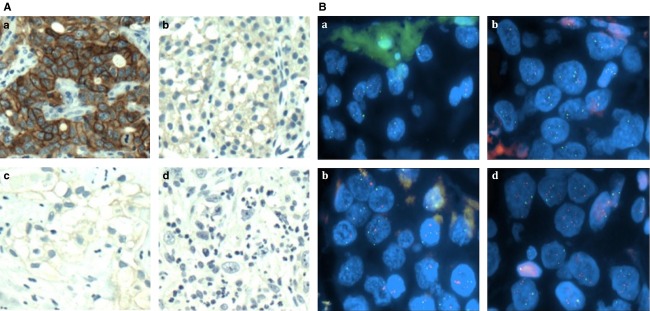
(A) HER2 expression in urothelial carcinoma. (a) HER2-positive staining scored as 3+, showing heavy membranous HER2 staining. (b) Moderate HER2 staining scored as 2+, demonstrating moderate membranous HER2 staining. (c) Weak HER2 staining, which demonstrates weak membranous staining which is scored as 1+. (d) HER2-negative urothelial cancer, showing no membranous HER2 expression, and scored as 0. (B) *ERBB2* status in urothelial carcinoma. (a) Normal, nonamplified (ratio = 1), (b) polysomic, nonamplified (ratio = 1), (c) amplified (ratio = 3.5), and (d) amplified (ratio = 5). HER2, human epidermal growth factor receptor 2.

Detailed laboratory methods can be found in Data S1. Tumor samples on TMAs in triplicate were analyzed for HER2 expression. For all cases, the assessment of HER2 was performed using the 2010 USCO/CAP HER2 guidelines as established for breast cancer 26, which has been previously employed to assess HER2 expression in bladder cancer 16,27. HER2 staining and its categorization based on localization and intensity is depicted in Figure 1A. All cases were scored for HER2 IHC status by a single pathologist (E. S.). For any sample with a score of less than 3+, status was validated by FISH.

### FISH

To assess the genetic status of *ERBB2*, FISH was performed on FFPE tissue from TMAs. Detailed laboratory methods can be found in Data S1. Assessments of ratios below 1.8 were considered negative and ratios more than 2.2 were considered positive for *ERBB2* gene amplification. Ratios varied between 1.8 and 2.2 were considered as equivocal. In these cases, 60 additional cells were analyzed by a second scorer to obtain a conclusive result. When the average number of chromosome 17 signal numbers exceeded 2.5 per cell, the case was considered polysomic. Representative examples of HER2 FISH are shown in Figure 1B.

### Copy number analysis

Copy number variation (CNV) was evaluated only in the Spanish cohort by aCGH. Detailed laboratory methods can be found in Data S1. CGH Analytics software version 3.4 (Agilent Technologies, Santa Clara, CA, US) was used to analyze the aCGH data. *ERBB2* copy number gain was determined as specimens with a log base 2 ratio greater than 0.9.

### mRNA analysis

Total RNA was extracted from tumor specimens following manufacturer's protocols (Ambion RecoverAll, Life Technologies, Grand Island, NY). mRNA transcript expression of HER2 was quantified using color-coded oligonucleotides, synthesized by NanoString Technologies, Seattle, Wa, US and hybridized to these transcripts. Transcripts were counted using the automated NanoString nCounter® system. Counts were normalized with the nSolver Analysis Software (version 1.0) in which mRNA expression was compared to internal NanoString Technologies, Seattle, Wa, US controls, several housekeeping genes (*ACTB*, *GAPDH*, *HPRT1*, *LDHA*, *PFKP*, *PGAM1*, *STAT1*, *TUBA4A*, *VIM*), and invariant genes (*ANGEL1*, *DDX19A*, *NAGA*, *RPS10*, *RPS16*, *RPS24*, *RPS29*) in UC. These invariant genes were identified by analyzing gene expression variances in several published datasets 28,29. Differential expression of HER2 status versus wild-type tumors was calculated with the edgeR package 30.

### Mutation status

For each sample, 100 ng of tumor-derived genomic DNA was subjected to whole genome amplification. Next, regions containing loci of interest were amplified using polymerase chain reaction and then mass spectrometric genotyping using iPLEX: Sequenom, San Diego, Ca, US chemistries was performed. An automated mutation-calling algorithm was performed to identify candidate mutations. Putative mutations were further filtered by a manual review and selected for validation using multibase homogenous Mass-Extend (hME) chemistry. Only mutations found in iPLEX and confirmed by hME were considered validated mutations. *ERBB2* hotspot mutations sequenced are listed in Table S1.

### Soft agar assays

NIH-3T3 cells (ATCC Cell Lines, Middlesex, UK) transfected with pBabe-puro constructs containing mutant *ERBB2* cDNAs were maintained in Dulbecco's Modification of Eagle's Medium (DMEM) (Cellgro/Mediatech, Manassas, Va, US) supplemented with 10% calf serum (Invitrogen, Life Technologies, Carlsbad, Ca, US). Soft agar assays were performed as described previously 31.

### Statistical analysis

Fisher's exact tests were used to measure associations between patient clinical characteristics and HER2 IHC or *ERBB2* FISH amplification. Since there is no standard scoring for HER2 in bladder cancer, we followed the protocol used for breast cancer: all specimens that scored either 3+ by IHC or a ratio of greater than or equal to 2.2 by FISH were considered positive. OS was defined as the time from start of treatment for metastatic disease to death or last follow-up.

Kaplan–Meier method was used to summarize the median OS, and Cox proportional hazard models were used to assess the associations of HER2 positivity and OS.

## Results

### HER2 status

Table 1 summarizes baseline patient characteristics for all patients with clinical information (Spanish *N* = 111; Greek *N* = 102). The number of patients with available HER2 status is lower because of tissue fall-off during antigen retrieval and/or hybridization procedures, age-dependent decrease in DNA integrity, or a lack of neoplastic tissue within the TMA sample. Similarly, aCGH data were indeterminate for 17 patients in the Spanish cohort, most likely due to inefficient hybridization as a result of fragment and crosslink-dependent decrease in DNA integrity.

**Table 1:** Patient clinical characteristics and outcomes for any patient with HER2 or clinical data

	Spanish (*N* = 111), *N* (%)	Greek (*N* = 102), *N* (%)
Eastern Clinical Oncology Group Performance Status (ECOG PS)		
0	40 (36)	57 (56)
1, 2	71 (64)	40 (39)
Missing	0	5 (5)
Visceral disease		
Yes	42 (38)	38 (37)
No	69 (62)	58 (57)
Missing	0	6 (6)
Complete response		
Yes	25 (23)	10 (10)
No	81 (73)	87 (85)
Unevaluable	5 (5)	5 (5)
Death		
Yes	57 (51)	66 (65)
No	54 (49)	33 (32)
Missing	0 (0)	3 (3)

HER2, human epidermal growth factor receptor 2.

**Figure 2:**
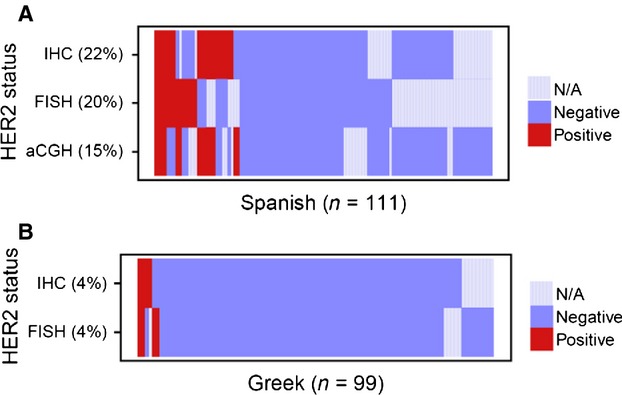
Heatmap of (A) Spanish cohort and (B) Greek cohort. Visualization of HER2 status based on different methodologies. N/A = data not available. HER2, human epidermal growth factor receptor 2.

*ERBB2* amplification by FISH was more frequent in the Spanish cohort compared to the Greek cohort (14/71 vs. 4/94, *P* = 0.006; Table 2). Similarly, IHC 3+ was more common in the Spanish cohort (19/88 vs. 4/93, *P* = 0.00058). The concordance rate between FISH and IHC was relatively low, indicated by a Kappa statistic of 0.47 (Table S2). These results are visualized in Figure 2.

**Table 2:** Frequency of IHC 3+, FISH amplification, and aCGH gain

	Spanish, *N* (%)	Greek, *N* (%)
FISH		
Normal	57 (80)	90 (96)
Amplified	14 (20)	4 (4)
IHC		
Negative (scored 0, 1+, 2+)	69 (78)	89 (96)
Positive (scored 3+)	19 (22)	4 (4)
aCGH		
Negative	80 (85)	
Positive	14 (15)	

IHC, immunohistochemistry; FISH, fluorescence in situ hybridization; aCGH, array-based comparative genomic hybridization.

### Survival analysis and association of HER2 status with clinical characteristics

**Figure 3:**
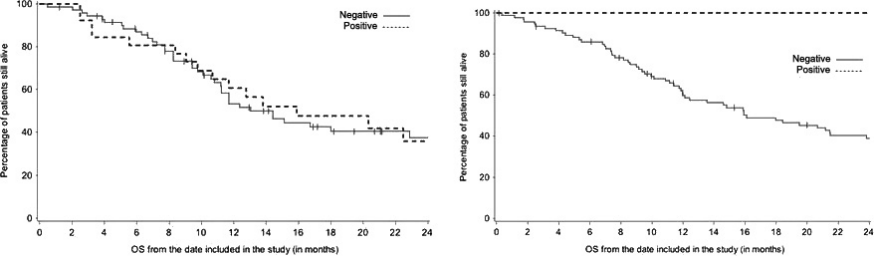
Overall survival by HER2 status for Spanish and Greek cohorts. No difference in overall survival was observed in the two cohorts. For the Spanish cohort, the hazard ratio was 0.94 (95% CI = 0.52–1.70, *P* = 0.83) and for the Greek cohort, the hazard ratio was 0.2 (95% CI = 0.03–1.48, *P* = 0.11). Multivariable analysis incorporating known prognostic factors showed similar nonsignificant results (Tables S2 and S3). HER2, human epidermal growth factor receptor 2.

Based on 3+ IHC and/or FISH ratio ≥2.2, 26 patients for Spanish and 5 patients for Greek cohorts were HER2-positive. Due to the differences between the rates of HER2 positivity, we analyzed the Spanish and Greek cohorts separately. We assessed the association of HER2 status and OS in both univariate and multivariate analysis and found no significant associations in either cohort (Fig. 3). We also tested associations between HER2 status and clinical characteristics and found no significant associations between prognostic variables or response and HER2 status in either cohort. Detailed result tables can be found in Tables S3A and S3B.

### Mutation hotspot sequencing

*ERBB2* mutations were identified at amino acid 755 and 777 in two (2%) patients in the Spanish cohort. These mutations were L755S and V777L. The specimen containing mutation L755S was also HER2-positive by IHC and copy number by aCGH, but HER2-negative by FISH. No HER2 IHC or FISH data for the specimen with mutation V777L were available.

### mRNA expression

**Figure 4:**
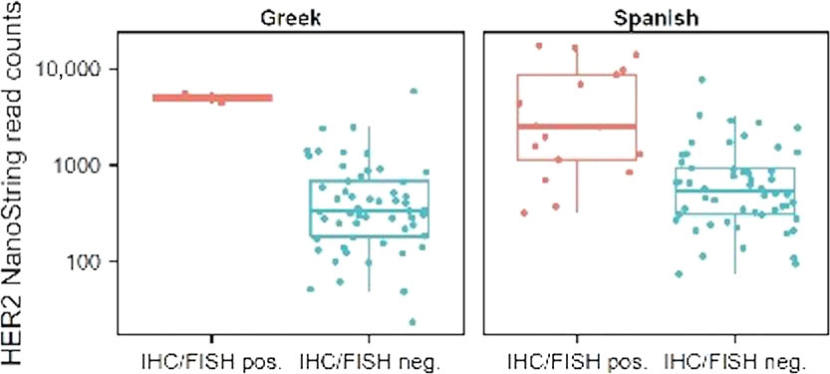
HER2 status and mRNA expression in Spanish and Greek cohorts.. The NanoString distributions of read counts HER2-positive and HER2-negative patients are visualized with box plots for both cohorts. HER2, human epidermal growth factor receptor 2.

HER2-positive tumors had increased levels of HER2 mRNA by NanoString in both the Spanish and Greek cohorts. The results are visualized in box plots in Figure 4.

### Functional analysis of HER2 V777L

Wild-type and mutant HER2 were ectopically expressed in murine NIH-3T3 cells and tested for oncogenic activity by assessing anchorage-independent proliferation in soft agar. Although HER2 V777L supported soft agar colony formation, two other mutants reported in the COSMIC database, V777A and V777M, did not (Fig. S1). HER2 C334S, a highly oncogenic extracellular domain mutant 7, was used here as a positive control. All HER2 mutants were expressed to similar levels, with the V777L mutant also exhibiting an increase in C-terminal phosphorylation (Fig. S2). These data are consistent with previous reports 32.

## Discussion

The impact of HER2 status on prognosis in metastatic UC has been controversial. To address the impact of HER2 status on survival in patients with metastatic UC, this study analyzed the primary tumors of two clinically characterized cohorts of 111 and 102 patients with UC that would later develop metastases using standard clinical tests, IHC and FISH. To further explore the pathway, we performed aCGH, although was only technically possible in one cohort. Between the two cohorts, we found that 16% of primary tumors demonstrated either IHC 3+ or FISH amplification. In addition, the concordance between FISH and IHC results was low, with many IHC-positive samples being FISH negative (Table S5). HER2-negative IHC staining demonstrated a high predictive value and specificity for negative *ERBB2* gene amplification, suggesting that it is a reasonable screening test for HER2 status in bladder cancer. IHC sensitivity for gene amplification is quite low (53%). We analyzed each cohort separately for clinical outcomes, and no significant associations between HER2 status and clinical outcomes were observed when controlling for known prognostic factors. While some clinical characteristics of the two cohorts were different, investigating both cohorts allows us to analyze the HER2 status across a large population of patients with metastatic UC.

The dependence of cancer cells on oncogenes for proliferation is well known. Whether HER2 is truly oncogenic in UC is not clear. However, we show that HER2 mRNA expression was increased in those tumors that were HER2-positive by IHC and FISH. These findings are similar to those found in HER2-positive breast cancer 33, suggesting that in fact the genomic and IHC findings in UC highlight an oncogenic dependence on the pathway in selected tumors.

HER2 copy number gains were also assessed by aCGH in the Spanish cohort as an exploratory analysis, and while there was significant overlap with the other modalities of assessing HER2, there were many specimens which were discordant (Fig. 3). Since aCGH integrates the results of all cells within a sample, it is not capable of distinguishing heterogeneity within a specimen, compared to FISH.

We expected to find similar frequencies of HER2 in both cohorts due to use of the same methodologies in the same laboratories and their similar clinical outcomes. Interestingly, there were significant differences between the Spanish and Greek cohorts, where 27% and 4% had HER2 overexpression and/or amplification, respectively. The large difference observed in these series suggests that HER2 status varies between populations, and raises the hypothesis that there is significant etiologic heterogeneity within bladder cancer that can lead to these differences. While we cannot rule out that subtle differences in fixation and storage could contribute to changes in HER2 antigenicity and *ERBB2* DNA, it is unlikely that these would affect DNA and proteins in the same manner.

Recently, Ross and colleagues reported the results of next-generation sequencing in 35 patients with UC. In this study, two (6%) patients presented genomic alterations in ERBB2: one patient with gene amplification and the other with mutation (S310F) 34. In addition, The Cancer Genome Atlas Project (TCGA) performed an integrated analysis to characterize molecular alterations in 131 patients with high-grade UC. This study identified mutations in 32 genes. Mutations or amplifications in *ERRB2* were also identified in 9% of patients 35. Interestingly, some of these molecular alterations are similar to those found in the TCGA for breast cancer, suggesting that these two tumors may share pathways for tumor progression. Interestingly, a high frequency (40%) of activating extracellular domain HER2 mutation has been detected in the infrequently found histological variant of micropapillary UC.

We identified a low frequency of *ERBB2* activating mutations in patients who developed metastatic UC. Not using next-generation sequencing might have overlooked the presence of some mutations. No patient in our series was described to have the micropapillary histological variant. Two mutations were identified by hotspot sequencing, both of which have been documented in other tumor types. HER2 L755S is a mutation identified in breast, gastric, colon, and lung cancers. In vitro testing indicates that this mutation confers resistance to lapatinib 32. In addition, HER2 V777L has also been documented as an oncogenic mutation in gastric and breast cancer, and remains sensitive to lapatinib 32. Although these molecular alterations have been identified in low frequencies, it may represent potential therapeutic targets in a specific subset of patients. In addition, future functional analysis of those mutations will be important to determine whether they represent targets for HER2-directed therapy.

While HER2 alterations do not lead to poorer outcomes in patients with advanced disease in this dataset, these findings do not exclude the utility of HER2 as a promising therapeutic target in UC. The presence of activating mutations in a small number of patients, as well as evidence of copy number gain and mRNA and protein overexpression, all suggest the importance of HER2 to the oncogenic phenotype of a subset of bladder cancers, and likely represents a therapeutic opportunity in a selected patients with locally advanced and metastatic UC.

Further work will be needed to ascertain the frequency of HER2 alterations in UC metastases and to examine the extent of HER2 concordance between primary and metastatic tissue, as there is some evidence that HER2 expression is increased in UC metastases 36. The addition of trastuzumab to cytotoxic chemotherapy in patients with evidence of HER2 expression was tested in a phase II study, and showed high levels of activity, although the study was designed to test safety and not efficacy 37. Single agent lapatinib was tested in unselected second line patients, and did not show significant evidence of anticancer activity 38. An ongoing randomized phase III study of maintenance lapatinib in UC patients with HER2 2 to 3+ IHC is ongoing (NCT00949455) and might shed additional light. Appropriately designed trials enriched for patients with HER2 alterations are needed to determine whether HER2-targeted therapies provide benefit.

The strengths of this study include the detailed clinical information available, the uniform treatment with platinum-based combinations (real-world use), the use of routine clinical tests to measure HER2 alterations (IHC and FISH), and findings of rare oncogenic mutations in bladder cancer. Limitations include the targeted rather than whole gene sequencing of *ERBB2*, which may underestimate the frequency of mutations and the relatively small sample size. In addition, we evaluated a selected group of patients which portend a poor prognosis and it can influence the association between HER2 status and survival outcomes in this population. Finally, we believe that exploratory analysis regarding clinical outcome or other clinical parameter will not produce reliable results. So, these correlations were not performed; thus, these results are hypothesis generating and require further external validation.

In summary, the impact of HER2 status on UC prognosis remains controversial. Our data suggest that neither IHC nor *ERBB2* gene amplification in the primary tumor play a role in prognosis after diagnosis of metastatic disease. However, the strong association between HER2 status and mRNA expression suggests that this pathway is active in selected cancers, and may yet represents a therapeutic target in UC.

## Supporting Information

**Figure S1.** NIH-3T3 soft agar assay. HER2 V777L leads to growth in soft agar compared to other V777 mutations. HER2 C334S was used as a positive control and the kinase-inactive mutant D845A was used as a negative control.

**Figure S2.** NIH-3T3 *ERBB2* western blot. HER2 V777L is associated with increased C-terminal phosphorylation.

**Table S1.** *ERBB2* mutation hotspots. **Table S2.** Concordance between FISH and IHC. **Table S3.** Association between HER2 status and OS from metastatic disease for (A) Spanish cohort and (B) Greek cohort. **Table S4.** Association of HER2 status with prognostic variables and treatment response. **Data S1.** Supplementary Methods

**Table S5.** Concordance IHC status versus FISH status.
