# Supplementary figures and images for: HER2 as a target in invasive urothelial carcinoma
Source: Cancer Med. 2015 Feb 26;4(6):844–52. doi: 10.1002/cam4.432 (PMC4472207; doi:10.1002/cam4.432)

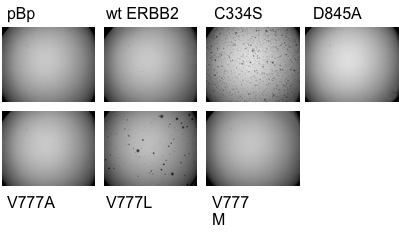

Supplement: Supplementary file 1 [file cam40004-0844-sd1.tif]

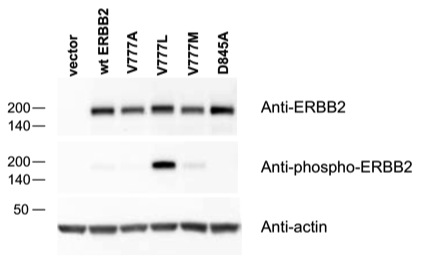

Supplement: Supplementary file 2 [file cam40004-0844-sd2.tif]
