# Supplementary material for: HER2 as a target in invasive urothelial carcinoma
Source: Cancer Med. 2015 Feb 26;4(6):844–52. doi: 10.1002/cam4.432 (PMC4472207; doi:10.1002/cam4.432)
Supplement: Supplementary file 3 [file cam40004-0844-sd3.docx]

**Supplementary Tables**

**Table S1: ERBB2 Mutation Hot-Spots**

| A775_G776insYVMA |
| --- |
| C334S |
| D326G |
| D769H |
| E321G |
| G776S |
| G776V |
| G776VC |
| I861fs |
| L755P |
| L755S |
| N319D |
| T216S |
| V750E |
| V777A |
| V777L |

**Table S2. Concordance between FISH and IHC**

|  | **FISH** | | | |
| --- | --- | --- | --- | --- |
| **IHC** |  | Not available | Negative | Positive |
|  | Not available | 0 | 17 | 2 |
|  | Negative | 28 | 122 | 8 |
|  | Positive | 6 | 8 | 9 |

**Table S3a. Association between HER2 status and OS from metastatic disease for Spanish Cohort**

| **Her2 status** | **N** | **Death** | **Median OS** | **HR and 95% CI** | **p-value** | **HR and 95% CI** | **p-value** |
| --- | --- | --- | --- | --- | --- | --- | --- |
|  |  |  |  | Univariate | 0.83 | Multivariate | 0.72 |
| Negative | 72 | 39 | 13 | 1 (reference) |  | 1 (reference) |  |
| Positive | 26 | 15 | 16 | 0.94 (0.52, 1.70) |  | 1.12 (0.61, 2.06) |  |

**Table S3b. Association between HER2 status and OS from metastatic disease for Greek cohort**

| **HER2 status** | **N** | **Death** | **Median OS** | **HR and 95% CI** | **p-value** | **HR and 95% CI** | **p-value** |
| --- | --- | --- | --- | --- | --- | --- | --- |
|  |  |  |  | Univariate | 0.11 | Multivariate | 0.12 |
| Negative | 95 | 65 | 16 | 1 (reference) |  | 1 (reference) |  |
| Positive | 5 | 1 | Not reached | 0.20 (0.03, 1.48) |  | 0.21 (0.03, 1.51) |  |

**Table S4. Association of HER2 status with prognostic variables and treatment response**

|  | Spanish Cohort | | | Greek Cohort | | |
| --- | --- | --- | --- | --- | --- | --- |
|  | HER2 | | P-value | HER2 | | P-value |
|  | Negative | Positive |  | Negative | Positive |  |
| Visceral metastasis |  |  | 0.64 |  |  | 0.65 |
| With | 28 | 12 |  | 37 | 1 |  |
| Without | 44 | 14 |  | 54 | 4 |  |
| ECOG PS |  |  | 0.48 |  |  | 0.65 |
| 0 | 24 | 11 |  | 53 | 4 |  |
| 1,2 | 48 | 15 |  | 39 | 1 |  |
| Complete Response |  |  | 0.29 |  |  | 0.49 |
| No | 50 | 22 |  | 82 | 5 |  |
| Yes | 19 | 4 |  | 9 | 1 |  |

**Supplementary Methods**

***Immunohistochemistry***

The Dana-Farber Cancer Institute Center for Molecular Oncologic Pathology preformed immunohistochemistry (IHC) to assess HER2 expression using a BioGenex i6000 automated staining platform (BioGenex Laboratories Inc., Fremont CA). Briefly, a 5µm FFPE section of a TMA was deparaffinized in xylene, followed by a graded alcohol rehydration. Antigen retrieval was performed by microwaving the tissue in citrate buffer three times for five minutes each time. HER2 antisera (Dako Inc., Carpentina, CA) was applied at 1:750 for 1 hour. Detection of the primary HER2 antibody was carried out using the BioGenex SS Polymer-HRP kit. Visualization of HER2 was accomplished using the DAB substrate kit (Vector Laboratories Inc., Burlingame, CA). The TMA was subsequently counterstained with hematoxylin, and then dehydrated in a graded series of alcohols prior to coverslip application.

***FISH***

To assess the genetic status of *ERBB2*, FISH was carried out on FFPE tissues. Slides derived from TMAs were deparaffinized in xylene, pretreated with EDTA solution in a microwave and digested with pepsin (0.05mg/ml in 0.01N HCL) for 30 minutes at 37ºC. After rinsing and air-drying, tissue morphology was assessed by phase contrast microscopy to ensure sufficient digestion of the collagen matrix. *ERBB2* probe mix (PathVysion, Abbott Molecular Inc, Des Plaines IL, USA) was applied to the slide. Pre-treated tissue sections and probe were co-denatured at 78ºC for 5 minutes and hybridized overnight at 37ºC in a hot plate (Hybrite chamber, Abbot Molecular Inc.). After post-hybridization washes, slides were counterstained with 4,6-diamino-2-phenilindole (DAPI counterstain) (Abbott Molecular Inc.). Results were analyzed in a fluorescent microscope (Olympus, BX51) using filter sets optimized for the PathVysion probe. Tissue sections were scanned at low magnification (100x) with DAPI excitation and a minimum of 60 nuclei were scored for *ERBB2*-to-CEP17 ratio.

***Copy number analysis***

Copy number variation was evaluated by array-comparative genomic hybridization (aCGH). Normal female 46, XX genomic DNA was obtained from Promega (Madison, WI). Genomic DNA was extracted using QIAamp DNA FFPE Tissue Kit (Qiagen, Valencia, CA). The Genomic DNA ULS labeling kit for FFPE Samples (Agilent) was used to chemically label 500ng of genomic DNA with either ULS-Cy5 (tumor) or ULS-Cy3 dye (normal/reference DNA) according to the manufacturer’s protocol (Agilent Technologies, Inc., Palo Alto, CA). Samples were hybridized to the Agilent SurePrint G3 Human CGH+SNP Microarray 4x180K. Each slide contains 4 identical arrays consisting of approximately 170,334 in situ synthesized 60-mer oligonucleotide probes that span coding and noncoding sequences with an average spatial resolution of 13 kb. The sample was applied to the array using an Agilent microarray hybridization chamber and hybridization was carried out for 40 hours at 65°C in a Robbins Scientific rotating oven at 20 rpm. The arrays were then disassembled according to the manufacturer’s protocol. Slides were dried and scanned using an Agilent DNA microarray scanner. CGH Analytics software version 3.4 (Agilent Technologies, CA) was used to analyze the aCGH data. *ERBB2* copy number gain was determined as specimens with a log base 2 ratio greater than 0.9.

Mutation Hot Spots

Interrogated mutation hot-spots can be found in Table 1. Primers and probes used for hME validation were designed using the Sequenom MassARRAY Assay Design 3.0 software, applying default multi-base extension parameters.

***Soft agar assays***

NIH-3T3 cells (ATCC) transfected with pBabe-puro constructs containing mutant *ERBB2* cDNAs were maintained in DMEM (Cellgro/Mediatech) supplemented with 10% calf serum (Invitrogen). Soft agar assays were performed as described.[^31^](#_ENREF_31) Briefly, 5x10^4^ cells were suspended in media containing 0.33% Select agar (Invitrogen) and plated on a bottom layer of media containing 0.5% Select agar in a 6-well plate. Plates were incubated at 37 ^o^C 2 weeks prior to imaging. For protein expression analysis, cells were lysed in a buffer containing 50 mM Tris-HCl (pH 7.4), 150 mM NaCl, 2.5 mM EDTA, 1% Triton X-100, and 0.25% IGEPAL CA630. Protease inhibitors (Roche) and phosphatase inhibitors (Calbiochem) were added prior to use. Immunoblotting was performed as described with anti-HER2 (Cell Signaling Technology #2242), anti-phospho-HER2 Y1221/1222 (Cell Signaling Technology #2243), or anti-actin (Santa Cruz Biotechnology #sc-1615).[^31^](#_ENREF_31)
