# Supplementary material for: HER2 as a target in invasive urothelial carcinoma
Source: Cancer Med. 2015 Feb 26;4(6):844–52. doi: 10.1002/cam4.432 (PMC4472207; doi:10.1002/cam4.432)
Supplement: Supplementary file 4 [file cam40004-0844-sd4.docx]

**Table S5. Concordance IHC status vs. FISH status**

**Greek Cohort:**

|  | IHC Status | | | |
| --- | --- | --- | --- | --- |
| Fish Status |  | Negative (0,1,2) | Positive (3) | Total |
|  | Negative (<2) | 84 | 1 | 85 |
|  | Positive (≥2) | 3 | 2 | 5 |
|  | Total | 87 | 3 | 90 |

| **Spanish Cohort:** | |  | |  | |  | |  | |
| --- | --- | --- | --- | --- | --- | --- | --- | --- | --- |
|  | IHC Status | | | | | | | | |
| Fish Status |  | | Negative (0,1,2) | | Positive (3) | | Missing | | Total |
|  | Negative (<2.2) | | 42 | | 7 | | 8 | | 57 |
|  | Positive (≥2.2) | | 6 | | 7 | | 2 | | 15 |
|  | Missing | | 12 | | 5 | | 0 | | 17 |
|  | Total | | 60 | | 19 | | 10 | | 90 |
|  |  |  |  |  |  |  |  |  |  |
